# Differential Severe Acute Respiratory Syndrome Coronavirus 2–Specific Humoral Response in Inactivated Virus–Vaccinated, Convalescent, and Breakthrough-Infected Subjects

**DOI:** 10.1093/infdis/jiad320

**Published:** 2023-08-12

**Authors:** Luisa F Duarte, Yaneisi Vázquez, Benjamín Diethelm-Varela, Valentina Pavez, Roslye Berríos-Rojas, Constanza Méndez, Claudia A Riedel, Jessica A White, Alexis M Kalergis, Susan M Bueno, Pablo A González

**Affiliations:** Millennium Institute on Immunology and Immunotherapy, Santiago, Chile; Departamento de Genética Molecular y Microbiología, Facultad de Ciencias Biológicas, Pontificia Universidad Católica de Chile, Santiago, Chile; Departamento de Ciencias Biológicas, Facultad de Ciencias de la Vida, Universidad Andres Bello, Santiago, Chile; Millennium Institute on Immunology and Immunotherapy, Santiago, Chile; Departamento de Genética Molecular y Microbiología, Facultad de Ciencias Biológicas, Pontificia Universidad Católica de Chile, Santiago, Chile; Millennium Institute on Immunology and Immunotherapy, Santiago, Chile; Departamento de Genética Molecular y Microbiología, Facultad de Ciencias Biológicas, Pontificia Universidad Católica de Chile, Santiago, Chile; Millennium Institute on Immunology and Immunotherapy, Santiago, Chile; Departamento de Genética Molecular y Microbiología, Facultad de Ciencias Biológicas, Pontificia Universidad Católica de Chile, Santiago, Chile; Millennium Institute on Immunology and Immunotherapy, Santiago, Chile; Departamento de Genética Molecular y Microbiología, Facultad de Ciencias Biológicas, Pontificia Universidad Católica de Chile, Santiago, Chile; Millennium Institute on Immunology and Immunotherapy, Santiago, Chile; Departamento de Genética Molecular y Microbiología, Facultad de Ciencias Biológicas, Pontificia Universidad Católica de Chile, Santiago, Chile; Millennium Institute on Immunology and Immunotherapy, Santiago, Chile; Departamento de Ciencias Biológicas, Facultad de Ciencias de la Vida, Universidad Andres Bello, Santiago, Chile; PATH, Seattle, Washington, USA; Millennium Institute on Immunology and Immunotherapy, Santiago, Chile; Departamento de Genética Molecular y Microbiología, Facultad de Ciencias Biológicas, Pontificia Universidad Católica de Chile, Santiago, Chile; Departamento de Endocrinología, Facultad de Medicina, Pontificia Universidad Católica de Chile, Santiago, Chile; Millennium Institute on Immunology and Immunotherapy, Santiago, Chile; Departamento de Genética Molecular y Microbiología, Facultad de Ciencias Biológicas, Pontificia Universidad Católica de Chile, Santiago, Chile; Millennium Institute on Immunology and Immunotherapy, Santiago, Chile; Departamento de Genética Molecular y Microbiología, Facultad de Ciencias Biológicas, Pontificia Universidad Católica de Chile, Santiago, Chile

**Keywords:** SARS-CoV-2, antibody responses, breakthrough, CoronaVac, vaccination

## Abstract

**Background:**

We sought to identify potential antigens for discerning between humoral responses elicited after vaccination with CoronaVac (a severe acute respiratory syndrome coronavirus 2 [SARS-CoV-2] inactivated vaccine), natural infection, or breakthrough infection.

**Methods:**

Serum samples obtained from volunteers immunized with CoronaVac (2 and 3 doses), breakthrough case patients, and from convalescent individuals were analyzed to determine the immunoglobulin (Ig) G responses against 3 structural and 8 nonstructural SARS-CoV-2 antigens.

**Results:**

Immunization with CoronaVac induced higher levels of antibodies against the viral membrane (M) protein compared with convalescent subjects both after primary vaccination and after a booster dose. Individuals receiving a booster dose displayed equivalent levels of IgG antibodies against the nucleocapsid (N) protein, similar to convalescent subjects. Breakthrough case patients produced the highest antibody levels against the N and M proteins. Antibodies against nonstructural viral proteins were present in >50% of the convalescent subjects.

**Conclusions:**

Vaccinated individuals elicited a different humoral response compared to convalescent subjects. The analysis of particular SARS-CoV-2 antigens could be used as biomarkers for determining infection in subjects previously vaccinated with CoronaVac.

## Teaser

Immunoglobulin G against severe acute respiratory syndrome coronavirus 2 membrane and nucleocapsid antigens, alone or combined, could serve as potential differential biomarkers for distinguishing CoronaVac-vaccinated breakthrough case patients from CoronaVac–vaccinated individuals with 2–3 doses or coronavirus disease 2019 convalescents.

Severe acute respiratory syndrome coronavirus 2 (SARS-CoV-2), the etiological agent of coronavirus disease 2019 (COVID-19), produces significant morbidity and mortality worldwide and has caused considerable strain on public health systems [1]. Since 2021, most countries worldwide have deployed massive vaccination campaigns that successfully curb the worst outcomes of COVID-19 [2, 3]. However, the immune responses elicited in convalescent individuals, the vaccinated, and those vaccinated and then infected (breakthrough case patients) remain to be studied in depth in coming years to clarify the serological immune components that account for protection against this virus and those that confirm infection after previous vaccination [4, 5].

The genome of SARS-CoV-2 encodes 4 structural proteins, namely spike (S), nucleocapsid (N), membrane (M), and envelope (E) proteins, as well as 16 nonstructural proteins (nonstructural proteins 1–16 [NSP1–NSP16]), along with several accessory proteins (open reading frame [ORF] proteins 3a, 3d, 6, 7a, 7b, 8, 9b, 14, and 10) that play key roles in immune evasion and the replication cycle of the virus [6, 7]. Among these proteins, the viral structural S protein is highly immunogenic, and the generation of binding and neutralizing antibodies against S protein during natural infection and after vaccination against SARS-CoV-2 has been the focus of extensive investigation [8–10]. Antibodies elicited against other SARS-CoV-2 proteins, such as those produced against virus-inactivated vaccines, are less known.

Whole inactivated SARS-CoV-2 virion-based vaccines account for nearly half of the total of doses administrated in the world population, with favorable safety and immunogenicity results [11]. The efficacy and effectiveness of this vaccine against COVID-19–related hospitalizations and deaths are significant [12], especially when immunity is reinforced with a booster dose [13–15]. Whole-virus vaccines have a variety of structural viral antigens, many of which are not present in messenger RNA–based or vector-based vaccines and thus may elicit immune responses that relate somewhat antigenically to natural infection with SARS-CoV-2 [16]. Given this, we hypothesized that natural infection, vaccination with inactivated virus, and breakthrough infection elicit differential humoral responses against SARS-CoV-2 proteins. We sought to identify these putative differences, which could eventually be used as biomarkers for the differential serodiagnosis in these individuals.

## METHODS

### Subjects and Samples

Blood samples from vaccinated individuals were obtained from volunteers recruited in the clinical trial CoronaVac03CL (clinicaltrials.gov NCT04651790), carried out in Chile starting in November 2020, and written informed consent was obtained from each volunteer. The study was approved by the sponsoring institution’s ethical committee (identification no. 200708006), each institutional ethical committee from the other sites, and the Public Health Institute of Chile (ISP Chile; no. 24204/20). Blood samples from naïve and convalescent individuals were obtained from the biobank of clinical samples within the PATH institution. Serum samples were obtained from the following groups: (1) preimmune: samples obtained at the moment of the first CoronaVac dose; (2) second dose + 2 weeks: samples obtained 2 weeks after the second dose; (3) second dose + 4 weeks: samples obtained 4 weeks after the second dose; (4) third dose + 4 weeks: samples obtained 4 weeks after a booster (third) dose of CoronaVac (6 months after the first dose); (5) breakthrough + 2 weeks: samples obtained from subjects vaccinated with 2 or 3 doses of CoronaVac and then naturally infected with SARS-CoV-2, from whom the samples were taken 2 weeks after a positive polymerase chain reaction result; and (6) breakthrough + 4 weeks: 4 weeks after a positive polymerase chain reaction result.

Thirty-five subjects were evaluated for the vaccinated group, 10 for the breakthrough case patients after the second dose and 7 for the breakthrough case patients after the third dose. Serum samples provided by PATH were grouped as follows: (1) naïve: samples from 10 subjects who were not exposed to the virus and that were obtained before 2020; (2) convalescents: blood samples collected longitudinally at 1, 2, 4, and 8 weeks after the symptom onset from 10 subjects who were naturally infected with SARS-CoV-2; and (3) 9 samples obtained from convalescent subjects that were classified with low, medium, and high titer against SARS-CoV-2. Because these samples were deidentified, demographic data, the time of sample collection, and the age of the subjects is not specified. The information regarding the groups, sample numbers, and demographic characteristics is summarized in Table 1.

**Table 1.** Sample Distribution and Demographic Characteristics of Sample Donors

Group	Time Points Evaluated	Sex of Sample Donor	Samples by Donor Sex, No.	Age Group of Sample Donor (y)	Samples by Donor Age Group, No.	Total Samples
Vaccinated	Preimmune; 2^nd^ dose + 2 wk; 2^nd^ dose + 4 wk; 3^rd^ dose + 4 wk	Female	22	18–59	13	35
				>60	9	
		Male	13	18–59	6	
				>60	7	
Convalescent	Longitudinal samples: 1, 2, 4, and 8 wk	Female	6	18–59	5	10
				>60	1	
		Male	4	18–59	3	
				>60	1	
	Unspecified^a^	Unknown	Unknown	Unknown	Unknown	9
Breakthrough after 2nd dose	2 and 4 wk	Female	3	18–59	3	10
				>60	0	
		Male	7	18–59	4	
				>60	3	
Breakthrough after 3rd dose	2 and 4 wk	Female	3	18–59	2	7
				>60	1	
		Male	4	18–59	3	
				>60	1	
naïve^a^	Before 2020	Unknown	Unknown	Unknown	Unknown	10

Samples obtained from Washington coronavirus disease 2019 COVID-19 biorepository with no information regarding demographic.

### Dot Blot

Dot blot analyses were performed by immobilizing recombinant proteins on a solid matrix and blotting with pooled serum from samples. As a positive control, all proteins (500 ng) were incubated with a conjugated anti-His Tag antibody (Supplementary Figure 1). Proteins evaluated in the analysis included ORF1a polyprotein (Invitrogen; RP-87701), ORF3a (LSBio; LS-G145920), ORF8 (LSBio; LS-G145919), NSP1 (R&D Systems; 10666-CV), NSP8 (R&D Systems; 10633-CV), NSP9 (R&D Systems; 10631-CV), NSP10 (R&D Systems; 10630-CV), NSP14 (R&D Systems; 10667-CV), E (Sino Biological; 40609-V10E3); and M proteins (Sino Biological; 40598-V07E). Detailed information regarding procedures is included in the Supplementary Methods.

### Enzyme-Linked Immunosorbent Assays

First, in-house indirect enzyme-linked immunosorbent assays (ELISAs) were performed to detect humoral immunity against nonstructural ORF1a (Invitrogen; RP-87701), ORF3a (LSBio; LS-G145920), ORF8 (LSBio; LS-G145919), NSP1 (R&D Systems; 10666-CV), NSP8 (R&D Systems; 10633-CV), NSP9 (R&D Systems; 10631-CV), NSP10 (R&D Systems; 10630-CV), and NSP14 (R&D Systems; 10667-CV) and structural N (R&D Systems; 10474-CV), M (R&D Systems; 10690-CV), and E (Sino Biological; 40609-V10E3) SARS-CoV-2 proteins by using a nondenaturing buffer as the coating solution. Based on the dot blot results using denaturing buffer as the coating solution, in-house indirect ELISAs were then performed to assess humoral immunity against E, ORF3a, and NSP8 SARS-CoV-2 proteins using the denaturing buffer (Supplementary Figure 2). Detailed information regarding the procedures is provided in the Supplementary Methods.

### Surrogate Virus Neutralizing Test

The neutralizing capacities of the antibodies in the samples of the individuals was evaluated using the SARS-CoV-2 surrogate virus neutralizing test kit from GenScript (catalog no. L00847-A). Assays were performed according to the manufacturer’s instructions and are detailed in the Supplementary Methods.

### Data Analysis

For each subject, the antibody titers were defined as specified in the Supplementary Methods. Statistical differences in geometric mean titer (GMT) values among all the different evaluated time points and proteins by group were assessed using a two-way analysis of variance with Tukey multiple comparison posttest. Comparisons between CoronaVac-vaccinated individuals and convalescents were assessed using the Kruskal-Wallis test with Dunn multiple comparison posttest. The significance threshold was set at α = .05. GraphPad Prism 9.0 software was used for data plotting, multiple logistic regression, and receiver operating characteristic (ROC) curve statistical analyses.

## RESULTS

### Kinetics and Extent of SARS-CoV-2–Specific Antibodies Responses in Convalescents, CoronaVac-Vaccinated Individuals, and Breakthrough Case Patients

**Figure 1.**
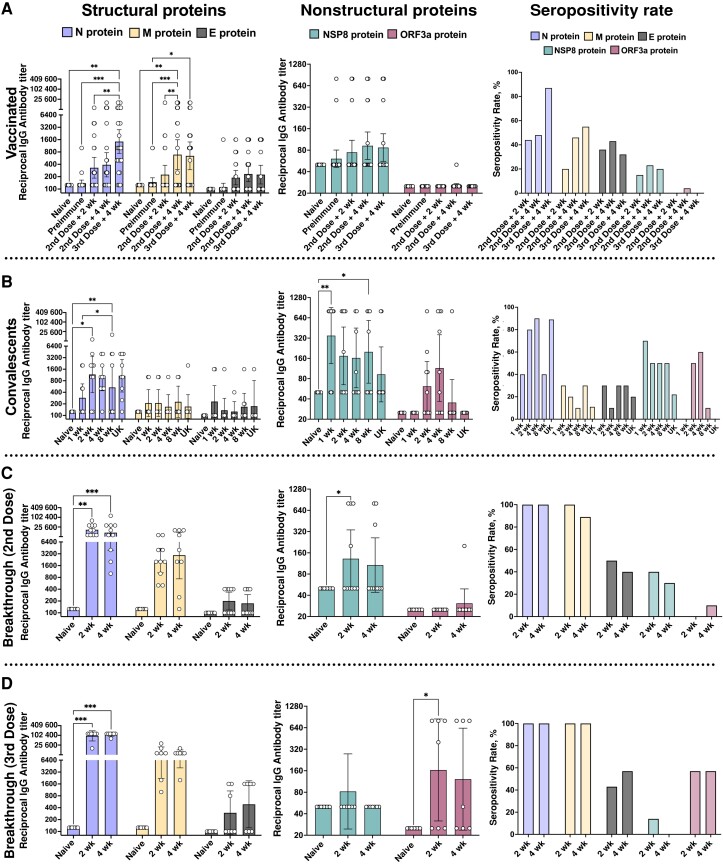
Kinetics of specific anti–immunoglobulin (Ig) G levels against multiple structural and nonstructural severe acute respiratory syndrome coronavirus 2 (SARS-CoV-2) proteins. *A*, Thirty-five serum samples from CoronaVac-vaccinated individuals were obtained at the moment of the first dose (Preimmune), 2 and 4 weeks after the second dose, and 4 weeks after a booster (third) dose. *B*, In 10en convalescent individuals, serum sampling was performed at 1, 2, 4, and 8 weeks after the onset of coronavirus disease 2019 (COVID-19) symptoms. In 9 convalescent individuals there was no information regarding the time of sample collection (unknown [UK]). *C*, Follow-up samples from 10 breakthrough vaccinated case patients who had received 2 doses of CoronaVac were obtained 2 and 4 weeks after a positive polymerase chain reaction (PCR) result. *D*, Follow-up samples from 7 breakthrough vaccinated case patients who had received a booster dose of CoronaVac (3 doses total) were obtained 2 and 4 weeks after a positive PCR result. Serum from 10 naïve individuals was added as controls*. Left panel,* Reciprocal antibody titers elicited against structural SARS-CoV-2 proteins (nucleocapsid [N], membrane [M], and envelope [E] proteins). *Middle panel,* Reciprocal antibody titers elicited against nonstructural SARS-CoV-2 proteins (nonstructural protein 8 [NSP8] and open reading frame [ORF] 3a protein). *Right panel,* Seropositivity rates for each protein at the respective time points. Bars represent geometric mean titers; error bars, 95% confidence intervals. A two-way analysis of variance test was used with Tukey multiple comparison posttest. *P < .05; **P < .01; ***P < .001.

We used indirect ELISA to evaluate the kinetics of the specific immunoglobulin (Ig) G antibodies against N, M, and E SARS-CoV-2 structural proteins and the nonstructural proteins ORF3a and NSP8, as these proteins showed some degree of differential immunoreactivity between the evaluated groups (Supplementary Figures 1 and 2). As expected, CoronaVac-vaccinated individuals showed a humoral immune response that was focused on structural components within the virion (Figure 1*A*). Remarkably, N-specific IgG antibody titers were significantly elevated only 4 weeks after administration of a booster dose (third dose), as compared with naïve controls, preimmune samples, and serum samples obtained 2 weeks after the second vaccine dose. M-specific antibodies titers significantly increased 4 weeks after the second dose, similarly to after the booster dose (Figure 1*A*). There were no significant antibody differences between vaccinated individuals and the negative controls for the NSP8 and ORF3a nonstructural proteins (Figure 1*A*). The highest antibody response in the vaccinated group was elicited against the N protein after the booster dose, which reached 87% seropositivity. In contrast, only about 50% of individuals had N-specific antibodies after the second vaccine dose or M-specific antibodies after 2 or 3 doses. On the other hand, approximately 20% of vaccinated individuals responded against the NSP8 protein (Figure 1*A*).

In convalescent individuals, compared with naïve controls, we detected significant differences in antibody titers when assessing reactivity toward the N and the NSP8 proteins (Figure 1*B*). Samples from convalescent individuals showed that the antibody response against the N protein was significantly higher compared with noninfected naïve individuals, starting 2 weeks after the onset of symptoms and continuing for up to 8 weeks (Figure 1*B*). Interestingly, we observed that the earliest antibody response against SARS-CoV-2 in the convalescent group was elicited against the NSP8 protein 1 week after onset of symptoms (Figure 1*B*). Among these proteins, the most immunogenic was the N protein, with seropositivity rates ranging from 80% to 90% at 2 and 4 weeks after onset of symptoms, respectively, followed by NSP8, which showed seropositivity rates of 70% and 50% after 1 or more weeks, respectively (Figure 1*B*).

Next, we investigated the specific-IgG antibody responses produced in individuals with symptomatic SARS-CoV-2 infection who were previously vaccinated with 2 or 3 doses of CoronaVac (breakthrough case patients) (Figure 1*C*and Figure 1*D* shows finding in breakthrough case patients after 2 and 3 vaccine doses, respectively). Here, the N-specific IgG antibodies showed the highest levels at both times evaluated, 2 and 4 weeks after COVID-19 diagnosis, and a 100% seropositivity rate. Although the antibody titers for the M protein did not reach significant levels compared with naïve controls, they showed high seropositivity rates of 89% and 100% in vaccinated individuals who had received 2 vaccine doses at 4 and 2 weeks after COVID-19 diagnosis, respectively (Figure 1*C*). Although to a lesser extent, we also found a significant difference between breakthrough case patients and naïve controls in antibody levels for the NSP8 protein after 2 weeks, with a seropositivity rate of 40% (Figure 1*C*).

Similar results were observed for breakthrough case patients who had received 3 vaccine doses, who reached seropositivity rates of 100% for both the N and M proteins (Figure 1*D*). In the case of the nonstructural proteins, a significant increase in antibody titers was seen for ORF3a at 2 weeks after COVID-19 diagnosis, with a seropositivity rate of 50% (Figure 1*D*). To harmonize the assessment of the humoral immune response to SARS-CoV-2 proteins, as recommended by the World Health Organization, we converted the results into binding antibody units (BAU) (Supplementary Figure 3 and Supplementary Table 1) [17]. Antibody values for other viral proteins than M, N, and NSP8 could not be reported as BAU owing to low levels of detection.

**Figure 2.**
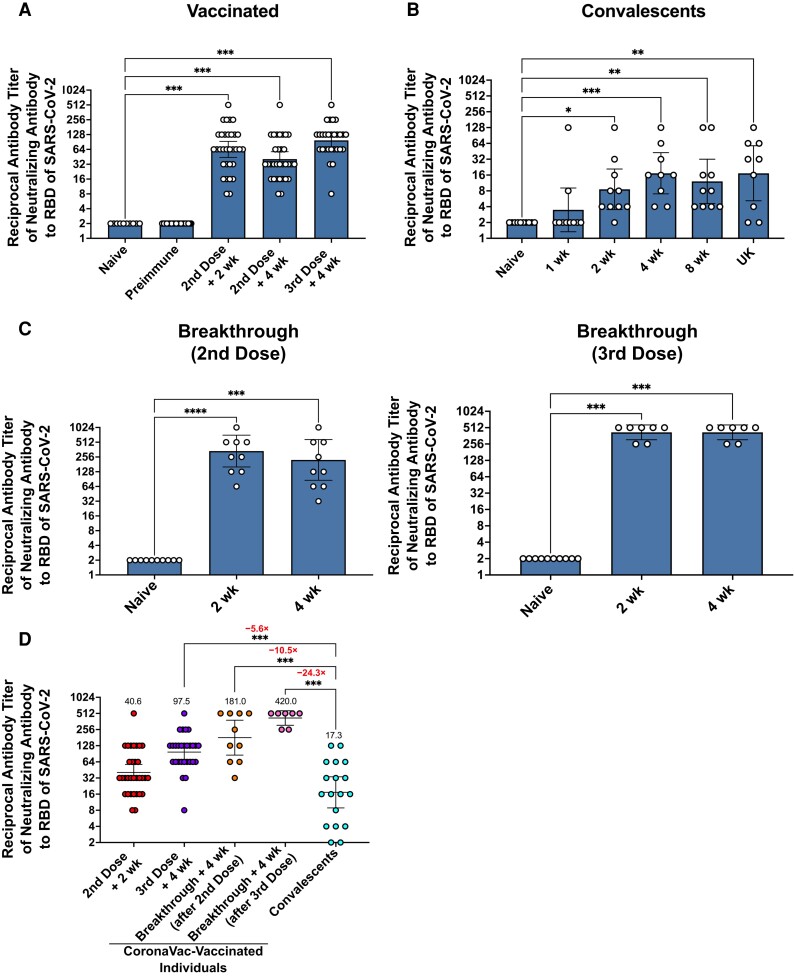
CoronaVac-vaccinated individuals show higher titers of neutralizing antibodies than nonvaccinated convalescent individuals. *A–C,* Neutralizing antibodies were evaluated using a surrogate virus neutralization test based on the interruption of human angiotensin-converting enzyme 2–spike protein interactions mediated by neutralizing antibodies for vaccinated subjects (*A*), convalescent individuals (*B*), and breakthrough case patients (*C*) after a second and third dose of CoronaVac. *D*, Comparison of the titers elicited in vaccinated individuals and in the convalescent group. Numbers above asterisks indicate fold variations for geometric mean titer values in the convalescent group compared with CoronaVac-vaccinated individuals. Data were analyzed using a Kruskal-Wallis test with Dunn multiple comparison posttest. Abbreviations: RBD, receptor-binding domain; UK, timing unknown. *P < .05; **P < .01; ***P < .001; ****P < .0001.

We also used a surrogate neutralization assay to determine the neutralizing capacity of antibodies in the serum of all groups evaluated against the receptor-binding domain (RBD) of SARS-CoV-2 (Figure 2) [18]. Figure 2*A* shows that CoronaVac-vaccinated individuals produced significant amounts of neutralizing antibodies 2 weeks after the second dose, with similar levels observed after administration of a third dose. Convalescent individuals also showed a significant increase in the levels of neutralizing antibodies against the RBD of S protein 2 weeks after symptom onset, which remained stable for up to 8 weeks (Figure 2*B*). Finally, breakthrough case patients showed the highest levels of neutralizing antibodies, being higher when natural infection occurred after the third dose of CoronaVac (Figures 2*C* and 2*D*).

### SARS-CoV-2-IgG Responses Among CoronaVac-Vaccinated Individuals, COVID-19 Convalescent, and Breakthrough Case Patients

To further investigate differences in the antibody responses between the different groups of individuals, we compared the GMTs elicited in each group and analyzed data from convalescent individuals with samples collected on unspecified dates and with samples collected 4 weeks after symptom onset. A summary of the GMT values and seropositivity rates obtained at these time points is shown in Table 2.

**Table 2.** Seroconversion Rates and Geometric Mean Titers of Antibodies Against Severe Acute Respiratory Syndrome Coronavirus 2 Proteins

Antibodies Detected	Indicators	2^nd^ Dose + 4 wk	3^rd^ Dose + 4 wk	Breakthrough + 4 wk (2 Doses)	Breakthrough + 4 wk (3 Doses)	Convalescents + 4 wk	Convalescents^a^
Anti-N IgG	GMT	389.58	1433.96	11313.71	115932.6	933.03	1080.06
	95% CI	197.27–769.37	736.33–2792.54	3951.77–32390.55	90986.72–147717.99	438.27–1986.35	406.66–2868.58
	Seropositivity rate, %	44	88	100	100	90	89
Anti-M IgG	GMT	688.50	643.33	2939.47	7245.79	168.24	170.10
	95% CI	298.68–1587.12	295.06–1402.67	734.28–11767.36	4069.99–12899.64	81.33–348.02	83.59–346.12
	Seropositivity rate, %	46	55	89	100	10	11
Anti-E IgG	GMT	232.03	224.49	174.11	487.61	125.99	174.11
	95% CI	151.12–356.25	133.11–378.60	104.33–290.56	123.83–1920.11	69.57–228.18	37.34–811.82
	Seropositivity rate, %	43	33	40	57	30	20
Anti-NSP8 IgG	GMT	92.31	87.06	107.18	50	162.45	92.59
	95% CI	59.26–143.80	55.80–135.82	44.08–260.61	50–50	58.57–450.01	36.18–236.97
	Seropositivity rate, %	27	24	30	0	50	22
Anti-ORF3a IgG	GMT	25.00	25.70	30.78	121.90	114.87	25.00
	95% CI	25.00–25.00	24.27–27.22	19.23–49.27	23.57–630.52	33.73–359.29	25.00–25.00
	Seropositivity rate, %	0	5	10	57	60	0

^a^Samples obtained from Washington coronavirus disease 2019 COVID-19 biorepository (timing unknown). Abbreviations: CI, confidence interval; E, envelope protein; GMT, geometric mean titer; Ig, immunoglobulin; M, membrane protein; N, nucleocapsid protein; NSP8, nonstructural protein 8; ORF3a, open reading frame 3a protein.

**Figure 3.**
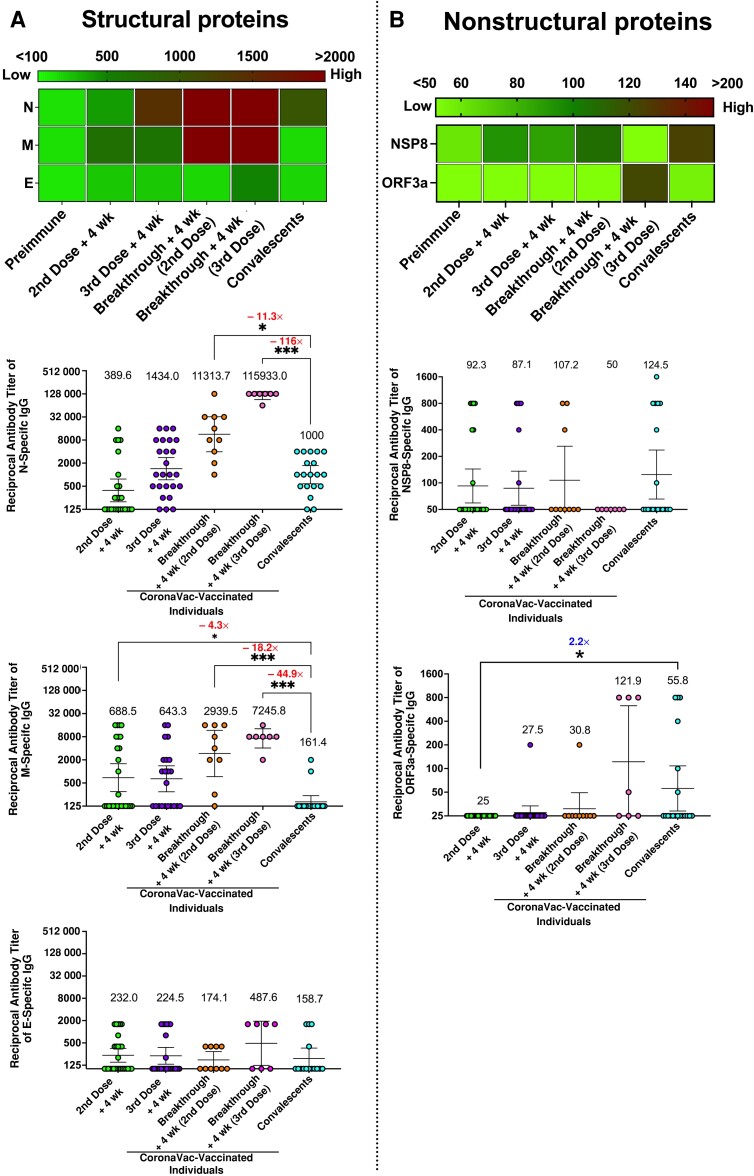
Comparison of antibody levels between convalescent subjects and CoronaVac-vaccinated individuals. Specific immunoglobulin (Ig) G antibodies titers in samples collected 4 weeks after the second or booster (third) dose in 26 individuals belonging to the vaccinated group and 4 weeks after the diagnosis of severe acute respiratory syndrome coronavirus 2 (SARS-CoV-2) infection based on positive polymerase chain reaction result in 10 and 7 breakthrough case patients who had received 2 or 3 vaccine doses, respectively. Individuals from the CoronaVac-vaccinated and breakthrough groups were compared with those from the convalescent group (samples obtained 4 weeks after the onset of symptoms as well as samples for which there are no data on when they were collected). *A*, Comparison of anti­–SARS-CoV-2 antibody levels against structural viral proteins using a heat-map display and reciprocal antibody titers against nucleocapsid (N), membrane (M), and envelope (E) protein–specific IgG, respectively. *B*, Comparison of anti–SARS-CoV-2 antibody levels for nonstructural viral proteins using a heat-map display and reciprocal IgG specific antibody titers against nonstructural protein 8 (NSP8) and open reading frame (ORF) 3a protein, respectively. Numbers above the groups represent geometric mean titers (GMTs); error bars, 95% confidence intervals. Numbers in red and blue indicate fold differences for GMTs in convalescent group compared with the CoronaVac-vaccinated groups. A Kruskal-Wallis test was used with Dunn multiple comparison posttest. *P < .05; ***P < .001.

Interestingly, the antibody responses were highest among breakthrough case patients for the structural proteins N and M, as shown in the heat map in Figure 3*A*. Significant differences of 11-fold and 18-fold were observed in the magnitude of the anti-N- and anti-M-specific IgG responses between breakthrough case patients who had received 2 vaccine doses and convalescent individuals. This IgG response increased to 116-fold and 45-fold for N- and M-specific antibody titers, respectively, when analyzing breakthrough case patients who had received 3 vaccine doses before infection. Importantly, convalescent individuals also had anti-M-specific IgG titers that were 4-fold lower than those in vaccinated individuals who received 2 vaccine doses (Figures 3*A*). No significant difference was observed in the overall levels of the anti-E­-specific antibody responses in vaccinated individuals compared with convalescents, suggesting that infection or vaccination alone does not induce significant antibody responses against this protein (Figure 3*A*).

Regarding the nonstructural proteins evaluated in this study, no significant differences were observed for the NSP8 protein among the different groups overall, although some individuals displayed high antibody titers, which potentially could relate to cross-reactivity with other coronaviruses (Figures 3*B*). Importantly, this response was not boosted after natural exposure to SARS-CoV-2. By contrast, we detected higher titers of ORF3a-specific antibody in convalescent than in CoronaVac-vaccinated individuals, but the highest response against this protein was elicited in breakthrough case patients who had received 3 doses of CoronaVac (Figure 3*B*).

Finally, we performed a ROC curve analysis to identify which of the evaluated SARS-CoV-2 proteins may better differentiate convalescents and CoronaVac-vaccinated individuals. Using this approach, the best performance was obtained when testing structural proteins and relating them with the number of vaccine doses administered to the subjects. High area under the ROC curve (AUC) values were obtained for the N protein (0.72 [95% confidence interval (CI), .56–.88]) and M protein (0.69 [.54–.85), when comparing CoronaVac-vaccinated individuals receiving only 2 doses and convalescents (Supplementary Figure 4*A*). However, the N protein showed a low AUC value (0.55 [95% CI, .38–.72]) when vaccinated individuals receiving the third dose were compared with convalescents. Moreover, the highest AUC value was achieved for the M protein when vaccinated individuals receiving the third dose were compared with convalescents (0.73 [95% CI, .57–.88]) (Supplementary Figure 4*B*).

As inactivated vaccines elicit a broad humoral immune response against various antigens, the serological diagnosis of previously vaccinated individuals and the evaluation of vaccine efficacy in a virus-exposed population is challenging. Next, we performed ROC curve analyses to identify SARS-CoV-2 proteins that may differentiate breakthrough case patients from vaccinated-only individuals and convalescents. As shown in Supplementary Figure 5, when the humoral immune response elicited in breakthrough case patients who had received 2 vaccine doses before infection is compared with that elicited in individuals vaccinated with 2 doses of CoronaVac, we found AUC values of 0.92 (95% CI, .83–1.0) for N, 0.71 (.54–.89) for M, 0.55 (.35–.75) for E, 0.52 (.30–.74) for NSP8, and 0.55 (.33–.77) for ORF3a protein, suggesting that M and N proteins could be useful for discriminating between both groups. We further compared individuals who received a booster (third) dose and found a similar tendency with the highest AUC values obtained for the N and M proteins, 0.83 (95% CI, .68–.97), and 0.76 (.58–.94), respectively. Remarkably, these proteins can also differentiate infected subjects without prior vaccination (convalescents) from breakthrough case patients (vaccinated, then infected), with AUC values up to 0.90 (95% CI, .77–1.0) for the N and 0.93 (.81–1.0) for the M protein (Supplementary Figure 5*A*).

In addition, we performed multiple logistic regression analyses to evaluate the capacity of the combined detection of N-specific and M-specific IgG antibodies to distinguish breakthrough case patients from the other groups evaluated. As shown in Supplementary Figure 5*B*, the combined data improved the AUC values obtained to 0.95 (95% CI, .88–1.0), 0.88 (.75–1.0), and 0.98 (.95–1.0), respectively. Importantly, this approach showed high negative and positive predictive powers of 92% and 80%, respectively, for differentiating breakthrough case patients from vaccinated individuals who received 2 doses and 90% and 73% for a comparison with subjects vaccinated with the booster dose and, finally, 90% and 89% for a comparison with convalescent individuals, suggesting that this may be a valuable strategy for serological diagnosis and evaluation of vaccine efficacy.

Scatterplots performed with hypothetical cutoffs of IgG antibody titers showed that it is possible to cluster the majority of the breakthrough case patients in the quadrant with the highest values for N and M antibody titers (cutoff, ≥1500 for M-specific or N-specific IgG antibody titers), compared with vaccinated-only individuals (2 or 3 doses), or infected-only subjects (convalescents) (Supplementary Figure 5*C*). Surprisingly, a ROC curve analysis showed that the immune response elicited against the N protein is sufficient to differentiate breakthrough case patients from vaccinated or convalescent individuals when these individuals are infected after receiving a booster dose, as the AUC values reached 1.0 in all the evaluated conditions (Supplementary Figure 6).

## DISCUSSION

A better understanding of the humoral immune response arising from either natural infection, vaccination, or infection after vaccination is paramount for the identification of correlates of protection against SARS-CoV-2. This information will also be useful for the development of new diagnostic tools that may help better determine the infection status of individuals, facilitating epidemiological surveying and the implementation of adequate public health strategies [19]. Because CoronaVac consists of whole inactivated SARS-CoV-2 virions, it is composed of a diverse set of structural viral antigens that could be presented to the immune system, and thus it is somewhat expected to elicit a humoral immune response that may mirror some aspects of natural infection [20, 21].

Overall, we found that CoronaVac induces a humoral immune response that includes high levels of N-specific and M-specific antibodies in an important percentage of vaccinated individuals, which was enhanced after natural infection in breakthrough case patients. Furthermore, our results suggest that the combination of N- and M-specific IgG antibody levels could be a reliable biomarker for differentiating CoronaVac-vaccinated and infected individuals from nonvaccinated and infected and CoronaVac vaccinated individual who were not infected and have mixed immunity resulting from infection and vaccination.

Although we did not analyze potential correlations between the elicited antibody responses and protection against COVID-19, previous reports suggest that hybrid immunity, or additional antigen exposure after natural infection before or after vaccination, is associated with a lower risk of reinfection [19] and a higher effectiveness in neutralizing new variants of concern [21–24], as well as reduced COVID-19 hospitalization compared with naturally acquired immunity after infection alone [25]. Interestingly, high levels of IgG against the N protein at the time of hospital admission have been correlated with worsened COVID-19 clinical courses [26], which could be attributed to increased interleukin 6 production mediated by N-specific IgG antibodies observed after COVID-19 cytokine storm [27]. On the contrary, high levels of IgG against the N protein, detected after COVID-19 recovery, have been associated with a long-term protective effect against reinfections [28]. The effect of other antibodies on the outcome of COVID-19 has not been fully explored to date [29].

On the other hand, antibody titers against the ORF3a protein seemed to be elicited almost exclusively in response to natural infection [30]. Noteworthy, only a fraction (50%) of the infected individuals responded to this antigen, which consequently may not represent an appropriate biomarker for confirming previous SARS-CoV-2-infection in vaccinated individuals. Hence, it may be worthwhile to consider other nonstructural proteins in future analyses. Interestingly, NSP8-specific antibodies were elicited in an important fraction of convalescent individuals at early time points (70% during the first week after symptom onset). Although some individuals in the vaccinated group responded to this antigen, it is unclear whether they were previously exposed to the virus before vaccination or whether it corresponds to potential antibody cross-reactivity against other antigens or coronaviruses.

On the other hand, our results complement findings reported for the SARS-CoV-2 inactivated virus vaccine BBIBP-CorV, for which it was shown that the N protein, NSP7, and S2–78 peptide may be used independently or in combination for the effective discrimination between individuals receiving 2 doses of this vaccine and convalescent subjects [8]. However, individuals vaccinated with 3 doses or breakthrough case patients were not included, and the M-specific IgG response was not investigated. Another study that combined the use of anti-ORF3b and anti-ORF8 antibodies showed that these were accurate serological markers for the detection of early and late SARS-CoV-2 infection [31], but additional studies evaluating their potential for differentiating convalescent and vaccinated individuals at different time points after vaccination or with different vaccines need to be performed. Moreover, the specific properties of the antibodies elicited after vaccination or natural infection, other than their neutralization capacity, should be considered in future studies to accurately identify correlates of protection against COVID-19.

Noteworthy, some limitations of our study include the rather reduced sample size used and the nonavailability of demographic data for some convalescent individuals, together with their clinical symptoms of COVID-19. However, having access to additional samples for the groups analyzed herein is at this time complex, as the availability of samples from nonvaccinated individuals exposed to SARS-CoV-2 is quite limited at present owing to high vaccination coverage against COVID-19 worldwide. Furthermore, a considerable proportion of the world population has now been exposed to SARS-CoV-2, due to its high circulation, without necessarily undergoing molecular diagnosis; thus, having access to samples from vaccinated non–SARS-CoV-2–exposed individuals is difficult at this time.

In addition, a considerable fraction of individuals who have been vaccinated and then exposed to SARS-CoV-2 may also have not undergone a formal diagnosis of viral infection (molecular diagnosis). Thus, at present, it is challenging to obtain samples from additional individuals who could be assigned to the different groups analyzed in our study. Nevertheless, if such samples were available, it would be interesting to confirm the findings reported herein using high-throughput analyses that consider a significant number of samples accompanied by the corresponding demographic information and details of the timing of the infection and virus strains/variants, as well as the severity of the reported symptoms for nonvaccinated convalescent individuals and breakthrough case patients.

## Supplementary Data

Supplementary materials are available at *The Journal of Infectious Diseases* online. Consisting of data provided by the authors to benefit the reader, the posted materials are not copyedited and are the sole responsibility of the authors, so questions or comments should be addressed to the corresponding author.

## Supplementary Material

jiad320_Supplementary_Data

## Notes

***Author contributions.*** Conceptualization: L. F. D., Y. V., B. D. V., C.A.R., J. A. W., A. M. K., S.M.B, and P. A. G. Visualization: C.A.R., J. A. W., A. M. K., S. M. B. and P. A. G. Methodology: L. F. D., Y.V., B. D. V., V. P., R. B. R., and C. M. Investigation: L. F. D., Y. V., and B. D. V. Funding acquisition: C. A. R., A. M. K., S. M. B., and P. A. G. Project administration: A. M. K., S. M. B., and P. A. G. Supervision: A. M. K., S. M. B., and P. A. G. Writing—original draft: L .F. D., Y. V., and B. D. V. Writing—review and editing: L. F. D., Y. V., B. D. V., V. P., R. B. R., C. A. R., J. A. W., A. M. K., S. M. B., and P. A. G. The order of the authors was decided based on contributions to the writing, editing, and reviewing of the manuscript. L. F. D. and Y. V. contributed equally to the manuscript and to the study conceptualization, methodology, and investigation.

***Acknowledgments.*** We are grateful to the CoronaVac03CL Study Group (see Supplementary Materials for full list of participants). Special thanks to Rami Scharf, Miren Iturriza-Gomara and Jorge Flores from PATH for their support on experimental design and discussion. We also thank the Vice Presidency of Research (VRI), the Direction of Technology Transfer and Development (DTD), and the Legal Affairs Department (DAJ) of the Pontificia Universidad Católica de Chile. We are also grateful to the administrative directions of the School of Biological Sciences (FCB) and the School of Medicine (FMed) of the Pontificia Universidad Católica de Chile for their administrative support.

PATH facilitated serum panel donations for this work. Samples used in this work were provided by the Microbial Pathogenesis Laboratory and Molecular Biomedical Immunology Laboratory at FCB-UC and the Everett Clinic and Bloodworks Northwest, accessed through the Washington COVID-19 Biorepository.

***Disclaimer.*** Under the grant conditions of the Bill & Melinda Gates foundation, a Creative Commons Attribution 4.0 generic license has already been assigned to the author-accepted manuscript version that might arise from this submission. The funders did not have any additional role in the study design, data collection and analysis, decision to publish, or preparation of the manuscript.

***Financial support.*** This work was supported by the Bill & Melinda Gates Foundation (https://www.gatesfoundation.org/; grants INV-021239 and INV-016821); the Agencia Nacional de Investigación y Desarrollo (ANID) (grants N°1190830, N°1170964, and N°1190864 to A. M. K., S. M. B., and P. A. G., respectively, through the Fondo Nacional de Desarrollo Científico y Tecnológico (FONDECYT); fellowship 21221163 to B. D. V. through the National Doctoral Scholarship; and postdoctoral FONDECYT grant N°3210473 to L. F. D.); the Millennium Institute on Immunology and Immunotherapy (Iniciativa Científica Milenio, ICM, grant ICN2021_045; former ICN09_016, P09/016-F to A. M. K., S. M. B., and P. A. G.); and the Innovation Fund for Competitiveness (grant FIC-R 2017 [BIP code 30488811-0] to A. M. K., S. M. B., and P. A. G.). Scientific-clinical studies that generated serum samples evaluated in this report (CoronaVac03CL, CoronaVac03CL booster dose, and CoronaVac03CL second booster dose) were supported by the Ministry of Health, Government of Chile; the Confederation of Production and Commerce, Chile; the Consortium of Universities for Vaccines and Therapies against COVID-19, Chile; the Chilean Public Health Institute; the Millennium Institute on Immunology and Immunotherapy; and Sinovac Life Sciences. Funding to pay the Open Access publication charges for this article was provided by the Bill & Melinda Gates Foundation.
