# Supplementary material for: Differential Severe Acute Respiratory Syndrome Coronavirus 2–Specific Humoral Response in Inactivated Virus–Vaccinated, Convalescent, and Breakthrough-Infected Subjects
Source: J Infect Dis. 2023 Aug 12;228(7):857–67. doi: 10.1093/infdis/jiad320 (PMC10547456; doi:10.1093/infdis/jiad320)
Supplement: jiad320_Supplementary_Data [file jiad320_supplementary_data.zip › Supplementary Table 1.docx]

| Supplementary Table 1. Geometric mean units (GMU) of antibodies against SARS-CoV-2 proteins | | | | | | | |
| --- | --- | --- | --- | --- | --- | --- | --- |
| ANTIBODIES DETECTED | **INDICATORS** | **2^ND^ DOSE**  **+ 4 WEEKS** | **3^RD^ DOSE**  **+ 4 WEEKS** | **BREAKTHROUGH**  **+4 WEEKS**  **(Two doses)** | **BREAKTHROUGH**  **+4 WEEKS**  **(Three doses)** | **CONVALESCENTS + 4 WEEKS** | **CONVALESCENTS UNSPECIFIED INFECTION TIME** |
| Anti-N IgG | GMU | 79.1 | 197.2 | 1224.0 | 28845.0 | 127.0 | 116.7 |
|  | 95% CI | 41.75- 150.2 | 96.7 –  402.1 | 495.4 –  3026 | 14233.0 –  58456.0 | 67.84 –  237.7 | 50.74 –  268.2 |
| Anti- M IgG | GMU | 246.1 | 163.9 | 586.0 | 1240.0 | 51.26 | 31.9 |
|  | 95% CI | 136.3 – 444.2 | 94.75 -283.4 | 234.1 –  1467 | 642.3 –  2394 | 27.53 –  95.46 | 15.92 –  63.89 |
| Anti-NSP8 IgG | GMU | 15.5 | 14.68 | 20.5 | 13.5 | 20.66 | 23.62 |
|  | 95% CI | 13.2-  18.31 | 13.0-  16.57 | 10.4-  40.4 | 13.5 –  13.5 | 12.5 –  34.11 | 10.0 –  55.59 |
