# Supplementary material for: Differential Severe Acute Respiratory Syndrome Coronavirus 2–Specific Humoral Response in Inactivated Virus–Vaccinated, Convalescent, and Breakthrough-Infected Subjects
Source: J Infect Dis. 2023 Aug 12;228(7):857–67. doi: 10.1093/infdis/jiad320 (PMC10547456; doi:10.1093/infdis/jiad320)
Supplement: jiad320_Supplementary_Data [file jiad320_supplementary_data.zip › supplementary_acknowledgments.docx]

## 1. Scientific-Clinical Teams related to the obtention, and processing of the samples used in this study.

**Center CL01: Áreas Ambulatorias Marcoleta - Pontificia Universidad Católica de Chile.**

Álvaro Miguel Rojas González, Maria Soledad Navarrete Bello, Constanza Belén Del Rio Solis, Dinely Valeska Del Pino Lavín, Natalia Elizabeth Aguirre Concha, Grecia Marly Salinas Escala, Franco Vega Farias, Acsa Raquel Salgado Ovalle, Thomas Quinteros, Marlene Ortiz, Marcela Puente, Alma Muñoz, Patricio Astudillo, Monique Nicole Le Corre, Katia Abarca.

**Center CL02: Clínica San Carlos de Apoquindo - Red de Salud UC-Christus**

Marcela Potin Santander, Juan Catalán Henríquez, Melan Peralta Kong, Consuelo Zamanillo Moreira, Nicole Keller Riquelme, Rocio Fernández Bustos, Sofia Aljaro Ehrenberg, Sofia López Colomba, José Tomas González, Tania Weil Valjalo, Luz Opazo Barrientos, Paula Muñoz Galeb, Inés Estay Puebla, Miguel Cantillana Morales, Liliana Carrera del Canto, Matías Masalleras Oyarzún.

**Center CL04: Clínica Los Andes - Universidad de Los Andes**

Paula Guzmán Merino, Francisca Aguirre Boza, Aarón Cortés Rojas, Luis Federico Bátiz, Javiera Francisca Pérez Velásquez, Karen Pamela Apablaza García, Lorena Yates Barsotti, María de los Ángeles Valdés Valdés, Bernardita Hurtado, Veronique Venteneul, Constanza Astorga.

**Center CL05: Clínica Alemana - Universidad del Desarrollo**

Paula Andrea Muñoz-Venturelli, Pablo Agustín Vial, Andrea Ingrid Schilling Redlich, Daniela Pavez Azurmendi, Inia Andrea Pérez Villa, Amy Lisa Riviotta, Francisca Gonzalez Mc Cowley, Francisca Pilar Urrutia Goldsack, Alejandra Isabel Del Río Weldt, Claudia Andrea del Carmen Asenjo Lobos, Bárbara Paulina Vargas Latorre, Francisca Valentina Castro Fuentes, Alejandra Patricia Acuña Rogel, Javiera Constanza Gúzman Cancino, Camila Alejandra Astudillo Griffiths.

**Center CL06: Hospital Clínico Félix Bulnes - Universidad San Sebastián**

Carlos M Pérez, Pilar Espinoza, Andrea Martínez, Marcela Arancibia, Harold Romero, Cecilia Bustamante, María Loreto Pérez, Natalia Uribe, Viviana Silva, Bernardita Morice, Marco Pérez.

**Center CL07: Hospital Dr. Gustavo Fricke - Universidad de Valparaíso**

Marcela González, Werner Jensen, Claudia Pasten, Ma. Fernanda Aguilera, Nataly Martínez, Camila Molina, Sebastián Arrieta, Begoña López, Claudia Ortiz, Macarena Escobar, Camila Bustamante, Marcia Espinoza, Angela Pardo, Alison Carrasco, Miguel Montes, Macarena Saldías, Natalia Gutiérrez, Juliette Sánchez.

**Center CL08: Hospital Carlos Van Buren- Universidad de Valparaíso**

Daniela Fuentes Hulse, Yolanda Calvo Toro, Mariela Cepeda Corrales, Rosario Lemus Manzur, Muriel Suarez Saavedra, Mercedes Armijo Rodríguez, Shirley Monsalves González, Constance Marucich Baeza, Cecilia Cornejo Beas, Ángela Acosta Palacio, Xaviera Prado, Francisca Yáñez, Marisol Barroeta Andrade, Claudia López García.

**Center CL09: Complejo Asistencial Dr. Sótero del Rio**

Paulina Donato Inostroza, Martin Lasso Barreto, María Iturrieta Meléndez, Juan Giraldo Paramo, Francisco Gutiérrez Valenzuela, María Acuña Schlegel, Ada Cascone Scarpati, Raymundo Rojas Araya, Camila Sepúlveda Contreras, Mario Alex Contreras, Yessica Campisto Sanhueza, Pablo González Sanhueza, Zoila Quizhpi Mejias, Mariella Lopez García, Vania Pizzeghello Salfate, Stephannie Silva Monsalve.

**Microbial Pathogenesis Laboratory and Molecular Biomedical Immunology Laboratory - Pontificia Universidad Católica de Chile**

Jorge A Soto, Bárbara M Schultz, Mariana Ríos, Felipe Melo-González, Nicolás MS Gálvez, Gaspar A Pacheco, Liliana A González, Daniela Rivera-Pérez, Catalina A Andrade, Gisela Canedo-Marroquín, Camila Covián, Daniela Moreno-Tapia, Farides Saavedra, Omar P Vallejos, Guillermo Hoppe-Elsholz, Alejandro Piña-Iturbe, Hernán Peñaloza, Claudia Orellana, Diane Leighton.

## 2. Members of the Independent Data Safety Monitoring Committee.

-Luis Delpiano, MD, Pediatric Infectologist, Hospital San Borja Arriarán, Santiago, Chile.

-Macarena Lagos, MD, Immunologist, Clínica Las Condes and Hospital Padre Hurtado, Santiago, Chile.

-Gloria Icaza, MD, Epidemiologist and Statistician, Universidad de Talca, Talca, Chile.

-Leonardo Chanqueo, MD, Adult Infectologist, Hospital San Juan de Dios, Santiago, Chile.

-Mónica Imarai, PhD, Universidad de Santiago, Santiago, Chile.
