# Supplementary material for: Differential Severe Acute Respiratory Syndrome Coronavirus 2–Specific Humoral Response in Inactivated Virus–Vaccinated, Convalescent, and Breakthrough-Infected Subjects
Source: J Infect Dis. 2023 Aug 12;228(7):857–67. doi: 10.1093/infdis/jiad320 (PMC10547456; doi:10.1093/infdis/jiad320)
Supplement: jiad320_Supplementary_Data [file jiad320_supplementary_data.zip › supplementary_figure_legends.docx]

**Supplementary figure 1.** **Anti-SARS-CoV-2 antibodies detected by dot blot.** (**A**) Five hundred nanograms (500 ng) of SARS-CoV-2 recombinant proteins (ORF1a, ORF3a, ORF8, NSP1, NSP8, NSP9, NSP10, NSP14, E (envelope), M (membrane)) were immobilized in nitrocellulose membranes using 2 µL of proteins prepared in 8 M Urea denaturant buffer. The membranes were blocked with 10% BSA and incubated overnight at 4°C with a sera pool obtained from control naïve individuals, convalescents (PCR (+) + 4w), CoronaVac-vaccinated with 2^nd^ dose + 2 weeks, and breakthrough (PCR (+) + 2 weeks) subjects (dilution 1/250). (**B**) As positive controls, all proteins (500 ng) were incubated with an anti-His Tag antibody conjugated with biotin. After incubation with sera, the membranes were treated with anti-human IgG-HRP. Finally, the membranes were incubated with an enhanced chemiluminescence Western blot detection system.

**Supplementary figure 2. Anti-SARS-CoV-2 antibodies detected by indirect ELISA under non-denaturing and denaturing conditions.** ELISA plates were activated with SARS-CoV-2 N, M proteins (100 ng/well in non-denaturing coating buffer), E protein (500 ng/well in denaturing coating buffer, or non-denaturing coating buffer), ORF3a protein (250 ng/well in denaturing coating buffer, or non-denaturing coating buffer), or NSP8 protein (200 ng/well in denaturing coating buffer, or non-denaturing coating buffer) for 1 h at 37°C, and blocked with 10% m/w non-fat milk overnight at 4°C. Afterward, plates were incubated with a pool of sera from naïve, convalescents (PCR (+) + 4w), vaccinated with 2^nd^ dose + 4w, or breakthrough (PCR (+) + 2w) subjects for 1 h at 37°C (1/250). Then, plates were incubated with anti-human IgG-HRP (BD #555788, 1/2,000), TMB substrate (BD, #555214) and absorbance (OD: optical density) was read at 450 nm.

**Supplementary figure 3.** **IgG level kinetics against structural and non-structural SARS-CoV-2 proteins expressed as WHO-binding antibody units (BAU).** (**A**) Thirty-five serum samples from CoronaVac-vaccinated individuals were obtained at the first dose (pre-immune), two and four weeks after the second dose, and four weeks after a booster dose for evaluating N-specific IgG antibodies M-specific IgG antibodies, and NSP8-specific IgG antibodies. (**B**) Ten serum sampling of convalescent individuals was performed at 1, 2, 4, and 8 weeks after recovery from SARS-CoV-2 infection. Nine convalescent individuals without information regarding the time of sample collection (UK: Unknown) were included for evaluating N-specific IgG antibodies, M-specific IgG antibodies, and NSP8-specific IgG antibodies. (**C**) Follow-up samples obtained from ten breakthrough cases that had received two doses of CoronaVac were obtained two and four weeks after a positive PCR result for evaluating N-specific IgG antibodies, M-specific IgG antibodies, and NSP8-specific IgG antibodies. (**D**). Follow-up samples obtained from seven breakthrough cases that had received a booster dose of CoronaVac (three doses total) were obtained two and four weeks after a positive PCR result for evaluating N-specific IgG antibodies, M-specific IgG antibodies, and NSP8-specific IgG antibodies. Sera from ten naïve individuals were added as controls. Bars show the Geometric Mean Units (GMU), and the error bars indicate 95% CI. A Kruskal-Wallis test was used with Dunn’s multiple comparison post-test, *p < 0.05, **p < 0.01, ***p < 0.001, ****p < 0.0001.

**Supplementary figure 4. ROC analyses of IgG responses to structural and non-structural SARS-CoV-proteins for comparing** **CoronaVac vaccinated group and convalescent group.** ROC analyses of IgG responses to the N (violet), M (yellow), E (blue), NSP-8 (green), and ORF3a (pink) proteins to compare vaccinated individuals after the administration of (**A**) two or (**B**) three doses of CoronaVac and convalescent subjects. Area under the curve (AUC) values are indicated in parenthesis with a 95% CI.

**Supplementary figure 5. ROC analyses of IgG responses against structural and non-structural SARS-CoV-2-proteins for comparing CoronaVac breakthrough cases in individuals that received two doses of CoronaVac with vaccinated individuals and convalescent individuals.** (**A**) ROC curves were constructed to identify SARS-CoV-2 proteins that can differentiate breakthrough cases from individuals that received two doses of CoronaVac (left), individuals receiving three doses (two in the primary schedule, plus one booster) of CoronaVac (middle), and convalescent individuals (right). Sera collected four weeks after recovery from infection, vaccination, and breakthrough cases were analyzed. For the convalescent group, nine individuals with an unspecified time of sample collection were included. Area under the curve (AUC) values are indicated in parenthesis with 95% CI. (**B**) AUC values, negative predictive power, and positive predictive power for the combinations of M and N antibodies titers to differentiate the breakthrough cases from individuals that received two doses of CoronaVac (left), individuals that received three doses of CoronaVac (middle), and Convalescent individuals (right). (**C**) Scatter plots distributions along with cutoff values for IgG responses based on optimal Youden values related to ROC curves: convalescent individuals (light blue), individuals vaccinated with two (green), or three (violet) doses, and breakthrough cases (orange).

**Supplementary figure 6. ROC analyses of IgG responses to structural and non-structural SARS-CoV-proteins for comparing CoronaVac breakthrough cases that had received a booster dose of CoronaVac with vaccinated individuals and convalescent individuals.** ROC analyses of IgG responses to the N (violet), M (yellow), E (blue), NSP-8 (green), and ORF3a (pink) viral proteins to differentiate breakthrough cases after a booster dose from (**A**) individuals that received two doses of CoronaVac, (**B**) individuals receiving three vaccine doses (two in the primary schedule, plus one booster) of CoronaVac, and (**C**) convalescent individuals. Area under the curve (AUC) values are indicated in parenthesis with 95% CI.
