# Supplementary material for: Differential Severe Acute Respiratory Syndrome Coronavirus 2–Specific Humoral Response in Inactivated Virus–Vaccinated, Convalescent, and Breakthrough-Infected Subjects
Source: J Infect Dis. 2023 Aug 12;228(7):857–67. doi: 10.1093/infdis/jiad320 (PMC10547456; doi:10.1093/infdis/jiad320)
Supplement: jiad320_Supplementary_Data [file jiad320_supplementary_data.zip › supplementary_methods.docx]

***Dot blot*:** To immobilize recombinant proteins on a solid matrix, 500 ng of each protein was diluted in denaturing buffer [20 mM phosphate buffer (mixture of monobasic and dibasic phosphate), 0.5 M NaCl, and 8 M urea)] and spotted onto a nitrocellulose membrane (in 2 μL volume) (Thermo Scientific). Membranes with spots were air-dried for 15 min and subsequently blocked with 10% BSA diluted in 0.05% Tween-20 in PBS, for 2h at RT. After incubation, the membranes were washed with 0.05% Tween-20 in PBS (twice). Next, the membranes were incubated overnight at 4°C with a sera pool from naive, convalescents (+ 4 weeks), vaccinated with a second dose + 2 weeks, and breakthrough (PCR (+) + 2 weeks) subjects. A sera pool was incubated in a dilution of 1/250 with 1% BSA diluted in 0.05% Tween-20 in PBS. As a positive control, all proteins (500 ng) were incubated with an anti-His Tag antibody conjugated with biotin at a dilution of 1:3,000 in 1% BSA diluted in 0.05% Tween-20 in PBS (Supplementary Figure 1). After incubation, the membranes were washed with 0.05% Tween-20 in PBS (three times x 5 min) and incubated for 1 h at RT with 1:2,000 anti-human IgG-HRP (1 mg/ml, BD) diluted in 1% BSA with 0.05% and Tween-20 in PBS. The membranes with an anti-His Tag-biotin antibody (Genscript, # A00613) were incubated with Streptavidin-HRP (1:6,000; Abcam #7403). Finally, membranes were washed with 0.05% Tween-20 in PBS (three times), once with PBS, and then incubated with an enhanced chemiluminescence western blot detection system (Femto, ECL, Thermo Scientific # 34094).

***ELISA assays*:** First, we performed ELISAs assays with SARS-CoV-2 proteins in their native state (non-denaturing conditions) against non-structural (ORF1a, ORF3a, ORF8, NSP1, NSP8, NSP9, NSP10, NSP14), and structural (N, E and M) recombinant viral proteins (data no shown). Because these results were inconclusive for the non-structural proteins, we implemented dot blot analyses with the above-mentioned proteins prepared in denaturing coating buffer, as detailed above. We then evaluated the kinetics of the specific-IgG antibodies against N (R&D systems, #10474-CV) and M (R&D systems, #10690-CV)-SARS-CoV-2 proteins with non-denaturing conditions, and E (Sino Biological, #40609-V10E3), ORF3a (LSBio, #LS-G145920) and NSP8 (R&D systems, #10633-CV)-SARS-CoV-2 proteins with denaturing conditions. Briefly, high-binding 96-well ELISA plates (Corning, #9018) were activated with 100 ng of N and M antigen dissolved in carbonate-bicarbonate buffer (Bioleged 1x, #421701) for 1 h at 37°C and blocked with 10% m/w milk in PBS 1X - Tween 20 (0.05%) overnight at 4°C. Denaturing buffer (20 mM monobasic and dibasic phosphate buffer, 0.5 NaCl, and 8 M urea) was used as a co-acting buffer to evaluate anti-ORF3a, anti-E, and anti-NSP8-SARS-CoV-2 (250 ng, 500 ng, and 200 ng, respectively). Afterward, the plates were incubated with sera from participants using serial dilution factors ranging from 1/250 to 1/16,000 (N and M) and 1/50 to 1/1,600 (E, NSP8, and ORF-3a proteins) for 1 h at 37°C (diluted 1% m/w milk in PBS 1X - Tween 20 (0.05%)). In parallel, a WHO standard curve (NIBSC code: 20/268) was performed from dilutions of 1/40 to 1/360. Then, the plates were incubated with anti-human IgG-HRP (BD, # 555788) for 30 min at room temperature in darkness. Finally, plates were resolved using commercial TMB (BD OptEIA, # 555214), consisting of a substrate mixture, which was incubated at room temperature for 15 min in darkness. This reaction was stopped using 2 N H_2_SO_4_, and the absorbance at 450 nm was read.

***Surrogate Virus Neutralizing Test:*** Serial dilutions of the serum were prepared and then incubated with the HRP-RBD reagent supplied in the kit for 30 min at 37ºC to allow the binding of neutralizing antibodies to S1-RBD. Sera and controls previously incubated with the HRP-RBD were added to the ELISA plate pre-coated with the human angiotensin-converting enzyme 2 (hACE2) protein and incubated for 15 min at 37ºC. After the incubation, samples were discarded, and plates were washed. Finally, a developing solution provided in the kit was added for 15 min at RT and then quenched with a stop solution also supplied. Plates were read at 450 nm in a microplate reader (Biotek, Ref. 1506021).

***Data analysis*:** For all ELISA assays, corrected OD values were calculated by obtaining the average between each subject’s two replicates and subtracting the corresponding subject’s blank (i.e., the OD measurement from the “inactivated” well) for each dilution factor. An absorbance cutoff was calculated for each dilution factor to establish the seropositivity threshold. For each subject, the antibody titer was defined as the highest dilution factor where the corrected OD was higher than the cutoff value for the corresponding dilution factor. The Geometric Mean of Titers (GMTs) was calculated using all subject titers for a given visit. A standard curve was used to plot the antibody responses against the M, N, and NSP8 proteins in the samples as binding arbitrary units (BAU), by using the WHO International Standard for SARS-CoV-2 antibody (NIBSC code: 20/268), which was prepared according to the manufacturer’s instructions. Data were analyzed using the log concentration transformed, and the final concentration for each sample was the average of the product of the interpolated BAU from the standard curve and the sample dilution factor required to achieve the OD_450nm_ value that fell within the linear quantitative range. Samples with undetermined concentrations at the lowest dilution tested were assigned the lower limit of quantification (28.6; 23.6; and 13.5 BAU for N, M, and NSP8, respectively). The inhibition rate of HRP-RBD binding in the neutralizing test was calculated as follows: [OD_450nm_ value of negative control -OD_450nm_ value of sample]/OD_450nm_ of negative control*100, and titers were reported as the reciprocal of the highest serum dilution required to achieve 30% of inhibition. Samples exhibiting less than 30% inhibitory activity at the lowest dilution tested (1:4) were assigned a titer of 2.
