# Supplementary figures and images for: Differential Severe Acute Respiratory Syndrome Coronavirus 2–Specific Humoral Response in Inactivated Virus–Vaccinated, Convalescent, and Breakthrough-Infected Subjects
Source: J Infect Dis. 2023 Aug 12;228(7):857–67. doi: 10.1093/infdis/jiad320 (PMC10547456; doi:10.1093/infdis/jiad320)

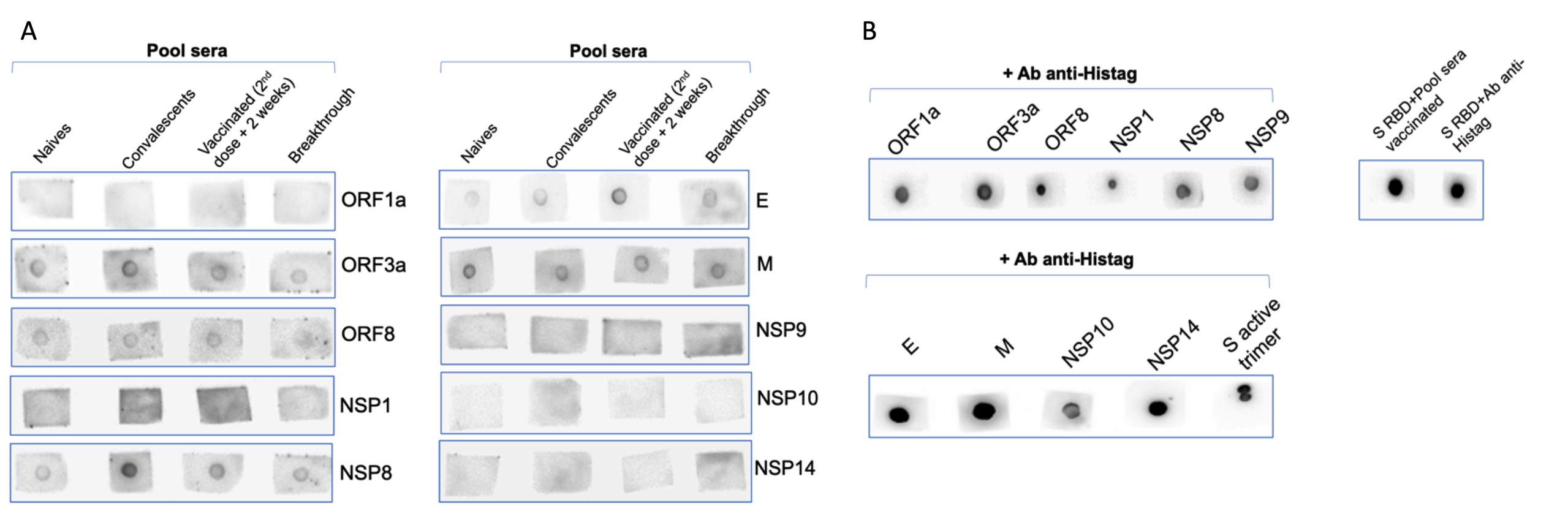

Supplement: jiad320_Supplementary_Data [file jiad320_supplementary_data.zip › supp_fig_1.tiff]

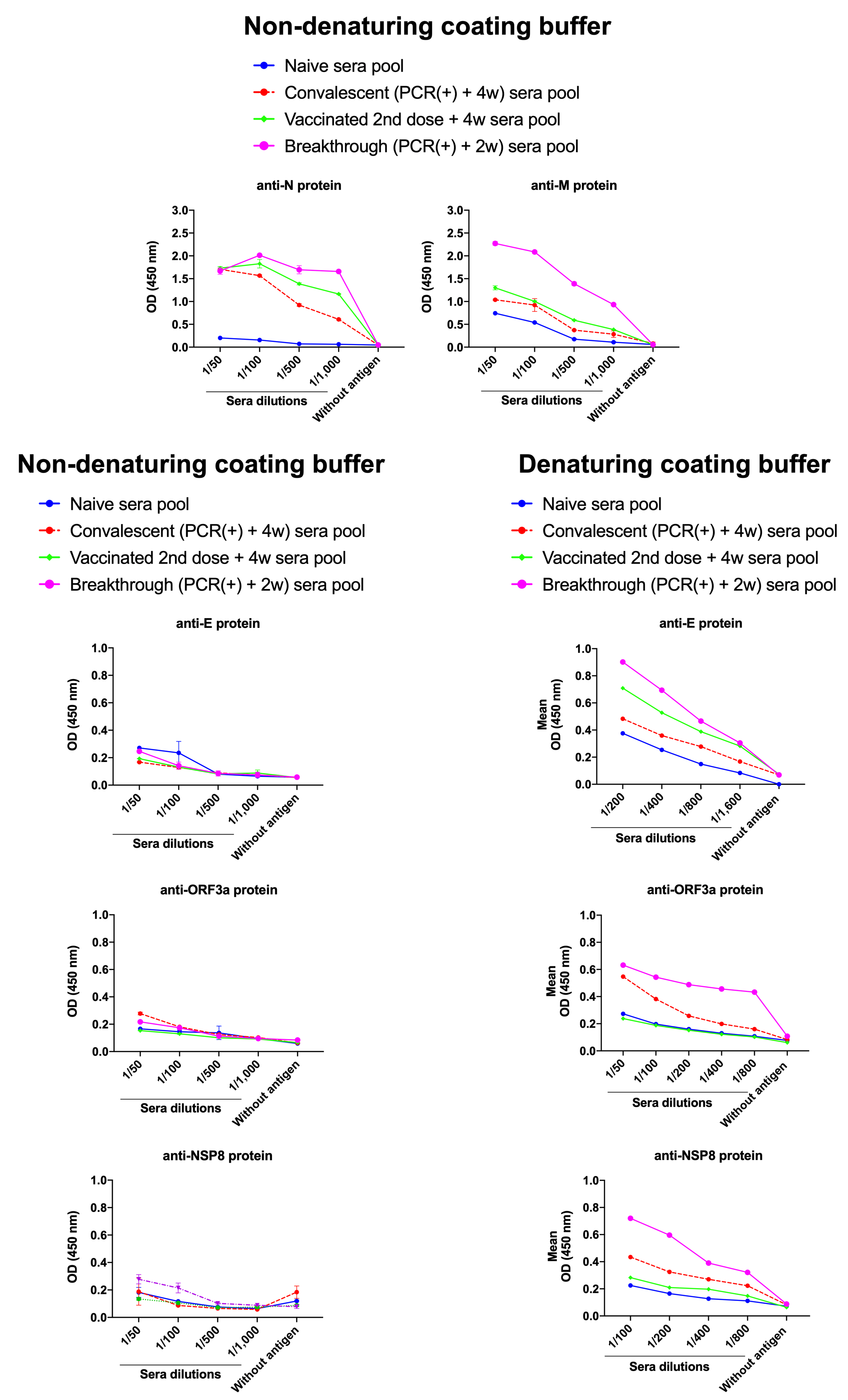

Supplement: jiad320_Supplementary_Data [file jiad320_supplementary_data.zip › supp_fig_2.tiff]

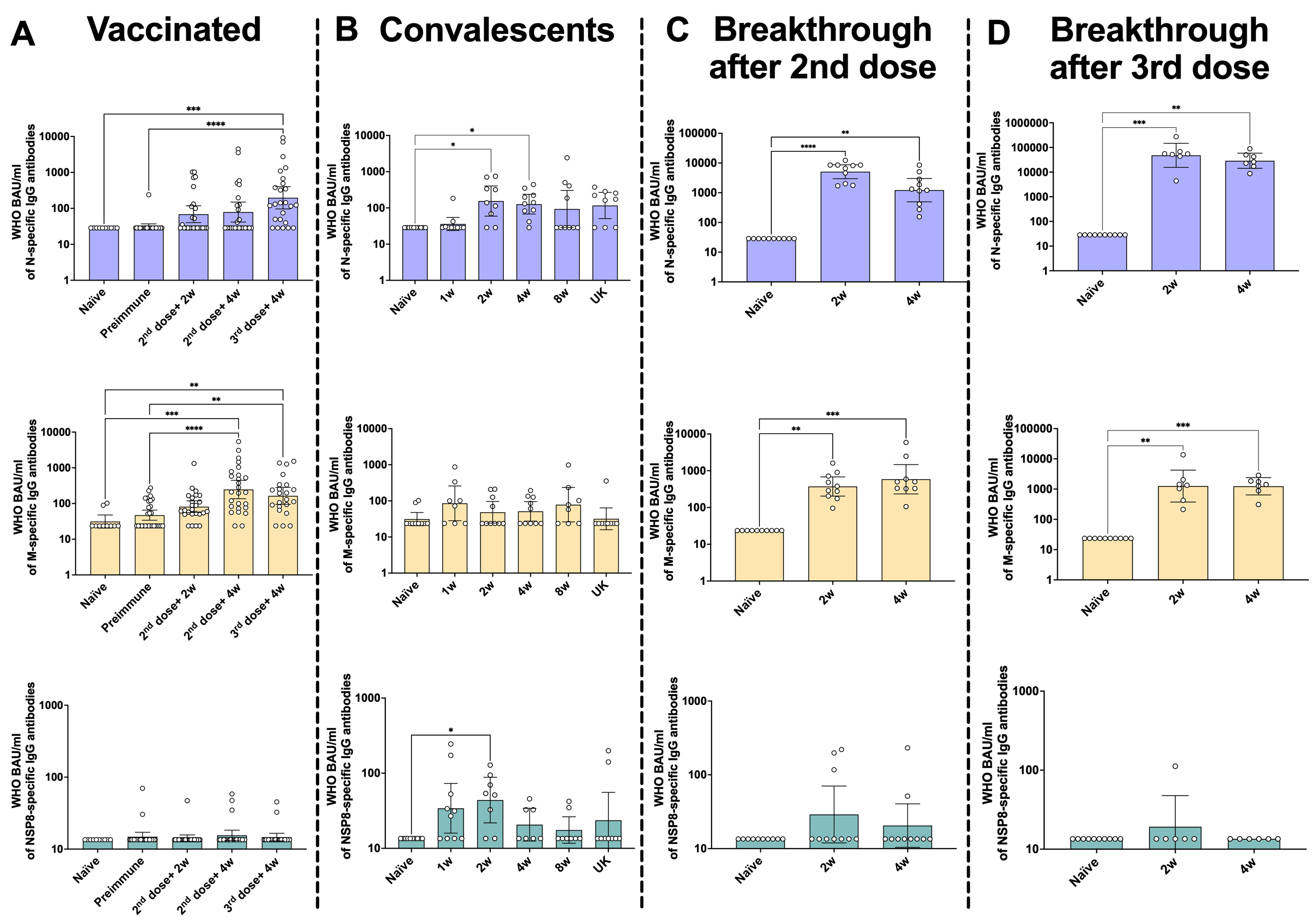

Supplement: jiad320_Supplementary_Data [file jiad320_supplementary_data.zip › supp_fig_3.tiff]

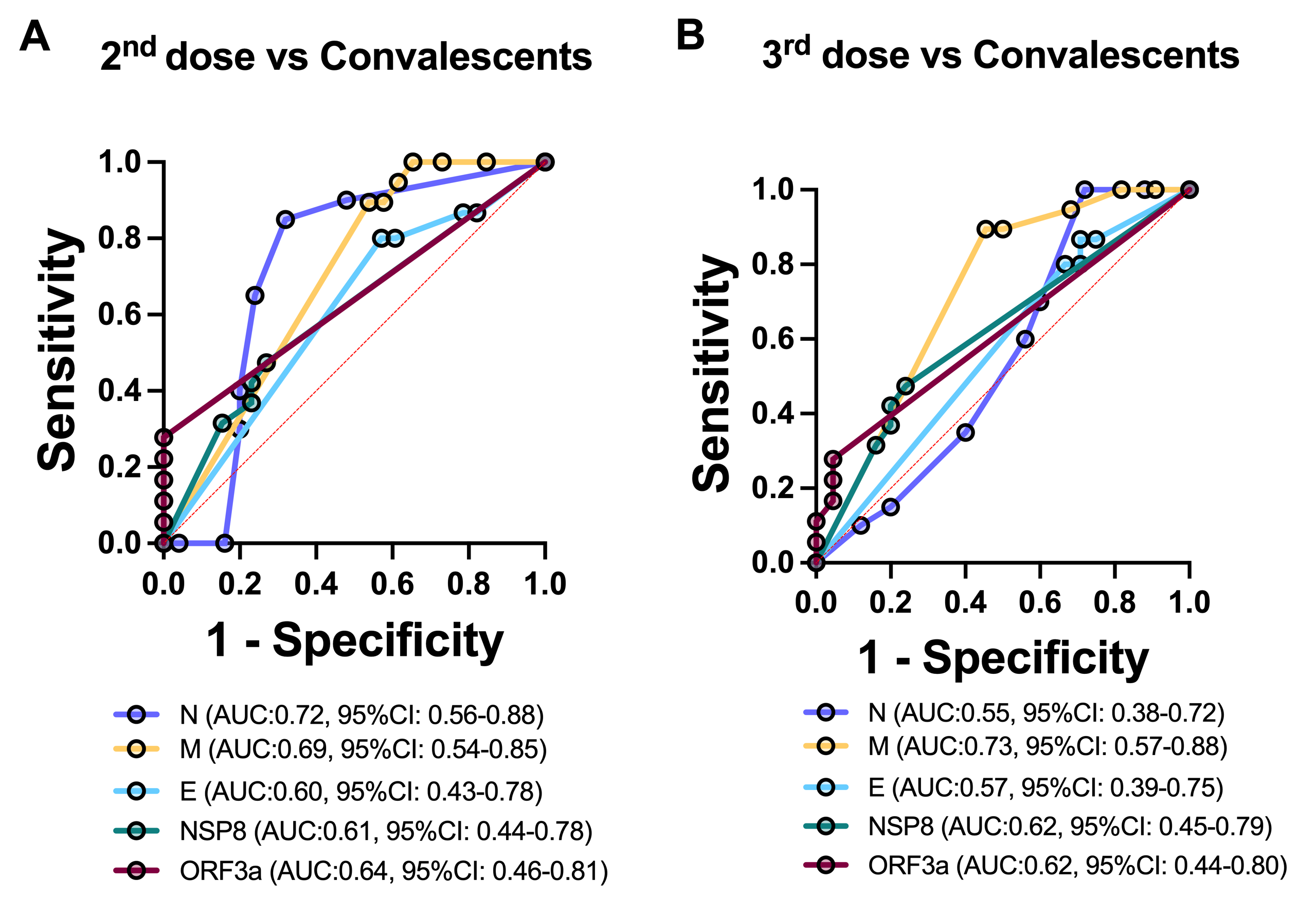

Supplement: jiad320_Supplementary_Data [file jiad320_supplementary_data.zip › supp_fig_4.tiff]

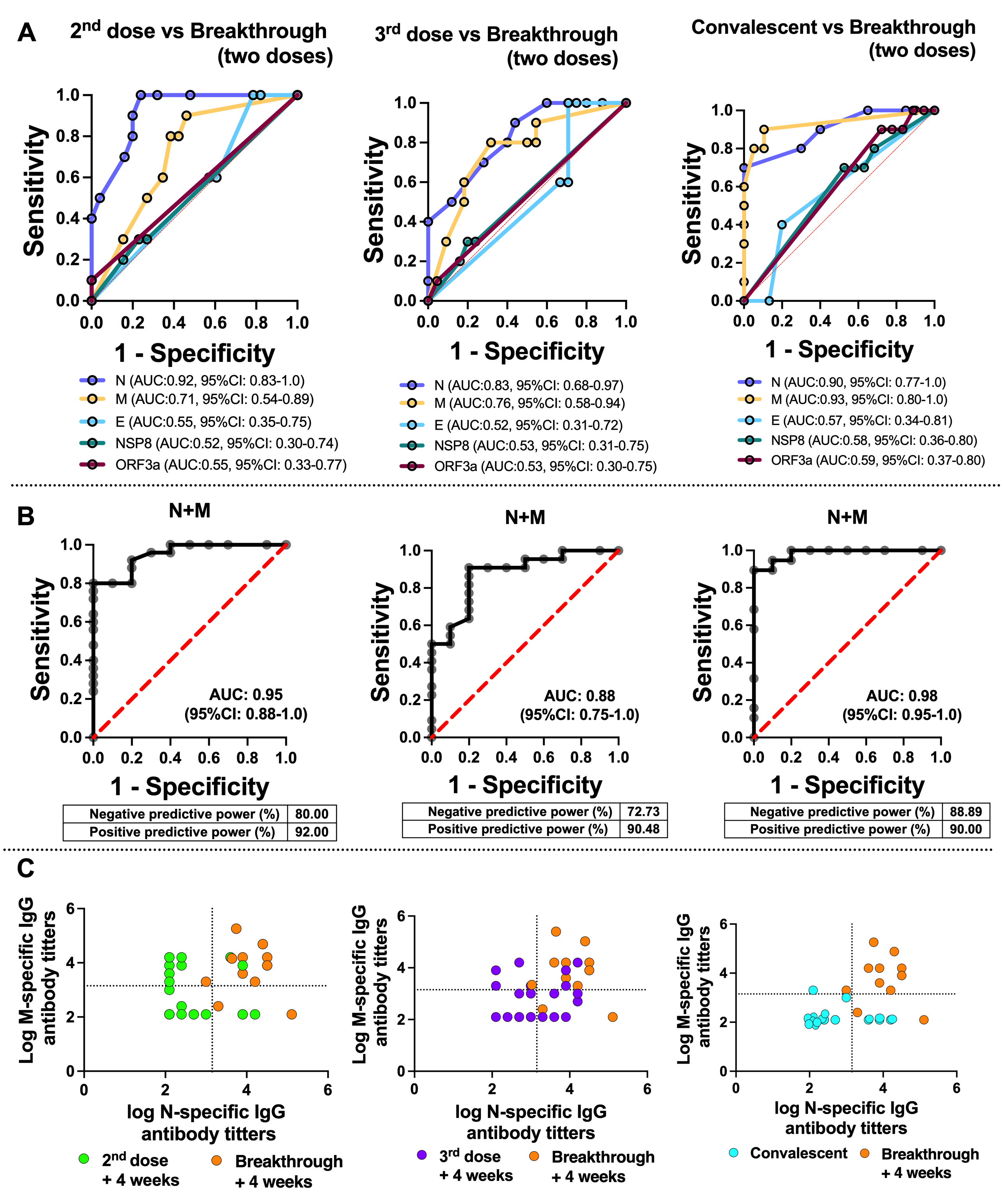

Supplement: jiad320_Supplementary_Data [file jiad320_supplementary_data.zip › supp_fig_5.tiff]

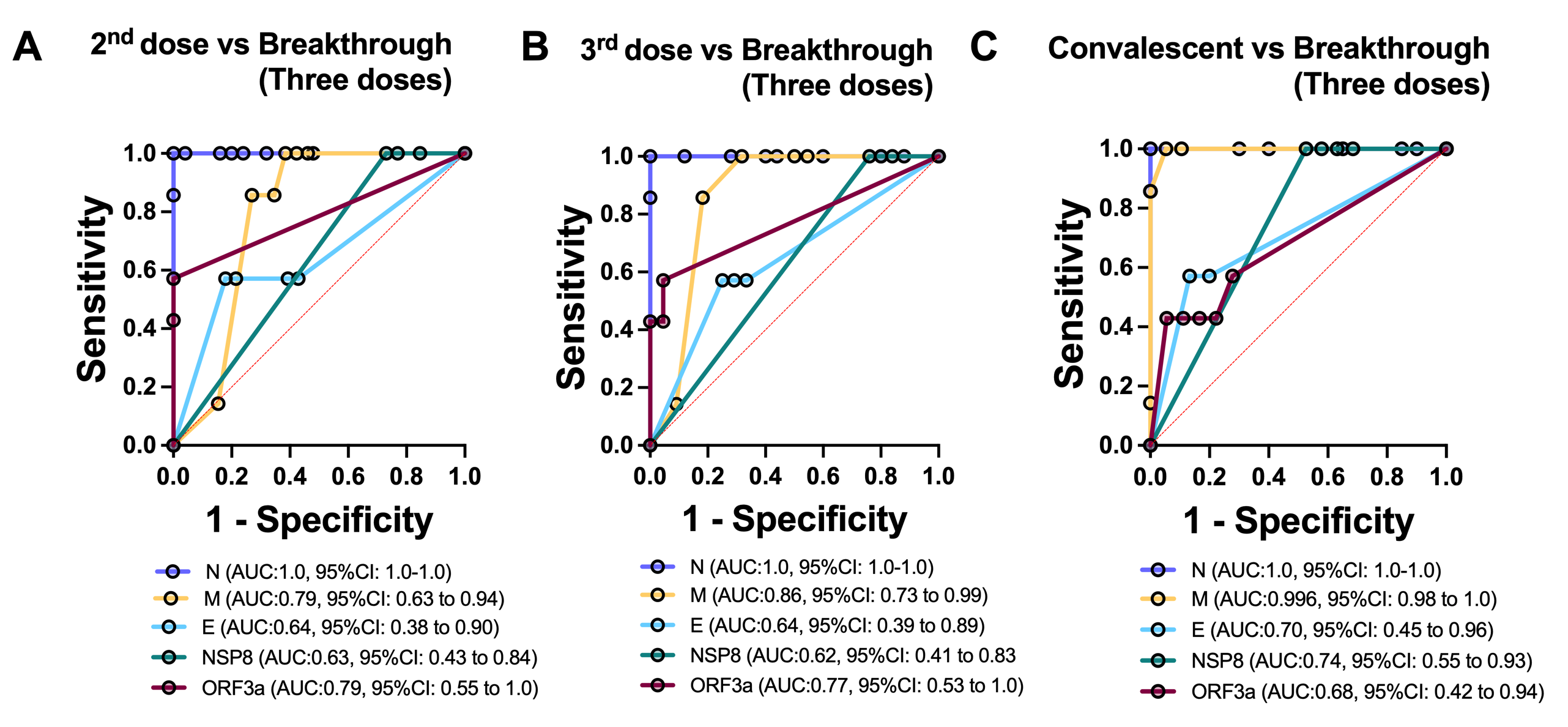

Supplement: jiad320_Supplementary_Data [file jiad320_supplementary_data.zip › supp_fig_6.tiff]
